# Familial lipoprotein lipase deficiency – neonatal presentation

**DOI:** 10.1186/1687-9856-2015-S1-P77

**Published:** 2015-04-28

**Authors:** Nip Siu-ying

**Affiliations:** 1 Prince of Wales Hospital, Hong Kong, China

## 

A baby girl, born by normal spontaneous vaginal delivery with birth weight 3.845kg, was admitted for neonatal jaundice on day 2 of life. Her mother had maternal gestational diabetes on diet control. Her father was newly diagnosed with hyperlipidaemia on health screen and was put on diet control. There was no consanguinity. Her serum bilirubin level on admission was 186mmol/L with normal liver function test. However, the serum was noted to be very lipaemic when checking with bedside bilirubin machine and it persisted for few blood samples. The baby girl was on exclusive breastfeeding. In view of lipaemic serum, random lipid profile was checked and showed hypertriglyceridemia (TG) 10.6mmol/L (<1.7mmol/L) and cholesterol (TC) 3.1mmol/L (<5.2mmol/L). Repeated fasting sample showed persistent elevated triglyceride level 8.5mmol/L. Other blood parameters including amylase and liver enzymes were unremarkable. Physical examination showed no dysmorphic features. There was no hepatosplenomegaly or xanthoma. Ophthalmologically examination did not show any lipaedmia retinalis.

In view of elevated serum triglyceride, further workup included lipoprotein electrophoresis and genetic analysis for lipoprotein lipase (LPL) gene were performed. Dietitian was consulted and the baby girl was given a therapeutic trial with commercially available special milk formula, Monogen (85% medium-chain triglyceride oil). The pretreatment lipid profile were TG 8.5mmol/L, TC 4.8mmol/L and HDL 0.6mmol/L (>1.3mmol/L) while TG 6.0mmol/L, TC 3.9mmol/L and HDL 0.6mmol/L after 2 weeks of special milk. She otherwise tolerated the special formula well.

Parents’ fasting lipid profiles were also checked. Mother showed hypertriglyceridemia 3.0mmol/L, and father had elevated TG 3.57mmol/L, TC 7.14mmol/L and LDL 4.65mmol/L (<2.6mmol/L). Lipid electrophoresis of baby girl detected a chylomicron band and a dense VLDL band, which was occasionally found in lipoprotein lipase (LPL) deficiency. Genetic analysis of LPL exon 6 by direct sequencing showed a heterozygous LPL NM_000237.2:c.835C>G and heterozygous LPL NM_000237.2:.836T>G mutations. Parental screening showed father carried a heterozygous LPL NM_000237.2:c.835C>G mutation while mother carried heterozygous LPL NM_000237.2:c.836T>G mutation. The patient was confirmed to be compound heterozygous lipoprotein lipase deficiency.

On monthly follow-up evaluations 10 months after hospital admission, the patient remained asymptomatic, maintained adequate growth, and had triglyceride levels gradually on downward trend 4.7mmol/l.

## Conclusion

This was a case of neonatal presentation of familial lipoprotein lipase deficiency, presenting with serum lipaemia.

Written informed consent was obtained from the patient for publication of this case report. A copy of the written consent is available for review by the Editor-in-Chief of this journal.

